# Small Molecule Drugs in Inflammatory Bowel Diseases

**DOI:** 10.3390/ph14070637

**Published:** 2021-06-30

**Authors:** Inès Ben Ghezala, Maëva Charkaoui, Christophe Michiels, Marc Bardou, Maxime Luu

**Affiliations:** 1 INSERM, CIC1432, Plurithematic Unit, 21079 Dijon, France; ines.ben-ghezala@chu-dijon.fr (I.B.G.); marc.bardou@u-bourgogne.fr (M.B.); 2 Clinical Investigation Center, Plurithematic Unit, Dijon Bourgogne University Hospital, 21079 Dijon, France; 3 Ophthalmology Department, Dijon Bourgogne University Hospital, 21079 Dijon, France; 4 Gastroenterology Department, Dijon Bourgogne University Hospital, 21079 Dijon, France; maeva.charkaoui@chu-dijon.fr (M.C.); christophe.michiels@chu-dijon.fr (C.M.)

**Keywords:** inflammatory bowel diseases, ulcerative colitis, Crohn’s disease, small molecule drugs

## Abstract

Inflammatory bowel diseases (IBDs), mainly represented by Crohn’s disease (CD) and Ulcerative Colitis (UC), are chronic disorders with an unclear pathogenesis. This incurable and iterative intestinal mucosal inflammation requires the life-long use of anti-inflammatory drugs to prevent flares or relapses, which are the major providers of complications, such as small bowel strictures and intestinal perforations. The introduction of tumor necrosis factor (TNF)-alpha inhibitors and other compounds, such as anti-IL12/23 and anti-alpha4/beta7 integrin monoclonal antibodies, has considerably improved the clinical management of IBDs. They are now the standard of care, being the first-line therapy in patients with aggressive disease and in patients with moderate to severe disease with an inadequate response to conventional therapy. However, for approximately one third of all patients, their efficacy remains insufficient by a lack or loss of response due to the formation of anti-drug antibodies or compliance difficulties with parenteral formulations. To address these issues, orally administered Small Molecules Drugs (SMDs) that use a broad range of novel pharmacological pathways, such as JAK inhibitors, sphingosine-1-phosphate receptor modulators, and phosphodiesterase 4 inhibitors, have been developed for CD and UC. This article provides an updated and complete review of the most recently authorized SMDs and SMDs in phase II/III development.

## 1. Introduction

Inflammatory bowel diseases (IBDs) refer mainly to Crohn’s disease (CD) and ulcerative colitis (UC). They are chronic, disabling diseases that may lead to severe medical and surgical complications and to extradigestive manifestations [1,2]. Several studies have shown an increasing worldwide incidence of inflammatory bowel diseases [3,4].

**Figure 1:**
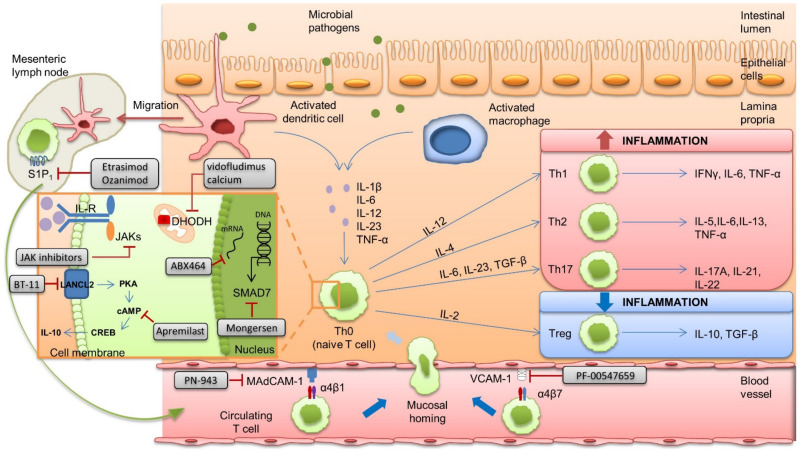
Presentation of the intestinal immune system in patients with inflammatory bowel disease and potential therapeutic targets for Small Molecule Drugs (SMDs). Intestinal immunity under normal conditions is regulated by both antigen-presenting cells (dendritic cells (DCs)) and macrophages in immediate proximity to DCs. Upon antigen recognition, activated macrophages and DCs produce proinflammatory cytokines that consequently promote the transformation of naive T and B cells into effector cells. In parallel, activated DCs migrate into the lymph node to promote the expression of gut homing integrins α4β7 and α4β1 on lymphocytes T and B. By interacting with MAdCAM and VCAM-1 in the digestive vascular compartment, lymphocytes expressing α4β7 or α4β1 enter the lamina propria. The immune response is regulated by balanced activity of anti-inflammatory Tregs and pro-inflammatory Th1, Th2, and Th17. In patients with inflammatory bowel disease, mucosal injury and inflammation lead to the epithelial barrier’s breach and invading intestinal microbial pathogens stimulate the proinflammatory immune response by activating dendritic cells (DCs) and macrophages. The balance between the anti-inflammatory activity of Tregs and the pro-inflammatory activity of Th1, Th2, and Th17 leans towards the latter, perpetuating local inflammation. Targets of SMDs are represented in this figure. cAMP, cyclic Adenosine Monophosphate; CREB, C-AMP Response Element Binding protein; DCs, dendritic cells; DHODH, Dihydroorotate Dehydrogenase; IFN, interferon; JAK, Janus Kinase; IL, interleukin; LANCL2, Lanthionine synthetase C–Like 2; MAdCAM, Mucosal Addressin Cell Associated Molecule; PKA, Proteine Kinase; S1P1, Sphingosine-1-Phosphate receptor 1; TGF, Transforming Growth Factor; Th0, naive T cell; Th1, T-helper-1 cell; Th2, T-helper-2 cell; Th17, T-helper-17 cell; Treg, regulatory T cell; TNF, Tumor Necrosis Factor; VCAM-1, Circulating Vascular Cell Adhesion Molecule-1. Illustration by Maxime Luu.

Current pharmacological options encompass several drug classes. While conventional therapies (aminosalicylates, corticosteroids, immunomodulators such as thiopurines, and methotrexate) are usually administered as the first treatment in cases of mild to moderate disease, biomolecular drugs developed in the last decade have completely reshaped the therapeutic strategy [5]. Biomolecular drugs are complex polypeptide chains with up to tertiary structures and a net higher molecular weight (mean: 150 kDa) [6]. Their goal is to induce and maintain clinical remission, prevent complications, and avoid long-term steroid use. In IBD, a step-up strategy is currently the standard of care: guidelines mostly advise the use of biomolecular drugs in moderate to severe IBD when first-step medications such as steroids, aminosalicylates, and immunomodulators have failed or are insufficient to induce or maintain remission. Nevertheless, a top-down strategy, that is, initial treatment with biologics or immunomodulators, is now considered, especially in patients with a complicated or extensive disease with poor prognostic factors [7,8,9]. Biomolecular drugs are usually required if conventional therapies fail and in severe diseases from the outset [10]. Biomolecular drugs are efficient to induce remission in IBD, but approximately one third of all patients experience a primary non-response, and 25 to 60% of initial responders experience a secondary non-response due to the appearance of anti-drug antibodies [11,12,13]. Moreover, the long-term use of biologics raises safety concerns, such as monoclonal-antibody-based TNF-alpha inhibitors (anti-TNF-α) and cancer [11,14,15]. In addition, biologics are administered intravenously or subcutaneously, which seems to affect the patients’ adherence and quality of life, especially for intravenous administrations [14,15]. Medication adherence and quality of life are key considerations in the therapeutic management of IBDs. Consequently, there remains a need for safer and more efficient treatments in IBDs. Small molecules drugs (SMDs) that use a broad range of novel pharmacological pathways, e.g., Janus kinases (JAKs) inhibitors, sphingosine-1-phosphate receptor modulators, and phosphodiesterase 4 inhibitors, have been developed for IBDs (Figure 1). SMDs are of low molecular weight (<1 kDa) and have no fixed structure. They are usually chemically opposed to biomolecular drugs. This difference in structure consequently affects their pharmacokinetics, mostly with a better diffusion capacity. Indeed, because of their small size, SMDs distribute easily through cell membranes, whereas the distribution of larger molecules is limited to the vascular or extracellular compartment. However, their short half-life requires oral intake daily or twice daily, whereas monoclonal antibodies can be administered monthly or every other month because of their slow catabolism. This short half-life is counterbalanced by a rapid onset of action [6]. Finally, the most significant advantage of SMDs may be the lack of immunogenicity, raising the hope of a sustained effectiveness. Nevertheless, oral administration leads to lower bioavailability and can be subject to hepatic or renal drug–drug interactions [16]. Herein, we review the most recently authorized SMDs and SMDs in phase II or III of clinical development.

## 2. Materials and Methods

We conducted extensive research, spanning from January 2016 to March 2021, on PubMed and clinicaltrials.gov (accessed on 18 May 2021) sites using the following terms: “inflammatory bowel diseases”, “Crohn’s disease”, “ulcerative colitis”, “therapy”, “clinical trials”, “interventional studies”, and “randomized clinical trials”. Research on clinicaltrials.gov (accessed on 18 May 2021) was filtered to keep only phase II trials or phase III randomized clinical trials (RCTs) and extension studies. The Pubmed search was filtered on studies evaluating SMDs regardless of design (i.e., RCTs, non-randomized controlled or uncontrolled trials, and observational studies). We focused our research on moderate to severe CD or UC in adults. Only studies published in English were retained.

## 3. Results

### 3.1. Janus Kinases Inhibitors

JAKs refer to four intracellular tyrosine kinase proteins: JAK1, JAK2, JAK3, and non-receptor tyrosine-protein kinase 2 (TYK2). Except for JAK3, which is specific to hematopoietic cells, the three other proteins are expressed ubiquitously. JAKs are activated in pairs through cytokine binding and, in turn, activate cytosolic DNA-binding proteins named signal transducers and activators of transcription (STATs). The JAK–STAT signaling pathway is implicated in hematopoiesis, adaptive and innate immunity, the inflammatory response, and the maintenance of the intestinal epithelial barrier’s integrity [17,18]. JAKs have been shown to be upregulated in IBDs [19,20,21]. For example, JAK1/JAK3 activation promotes B- and T-cell function and Th2 and Th17 differentiation. JAK1/JAK2/TYK2 are involved in B- and T-cell function, wound healing, and the antiviral response. JAK2 is involved in hematopoiesis and its inhibition may lead to cytopenia [19]. JAK1/JAK3/TYK2 are notably activated by IL-13 and are involved in the Th2 anti-inflammatory response, the epithelial barrier function, and B-cell activation. Il-13 is notably associated with impairment of the intestinal barrier function [22]. JAKs have therefore become a therapeutic target in IBDs. Unlike monoclonal antibodies, JAK inhibitors are able to target multiple cytokines and multiple inflammatory pathways [20,21]. Several JAK inhibitors with different selectivity and safety profiles are currently in use, all orally administered (Table 1).

#### 3.1.1. Tofacitinib

Tofacitinib is a pankinase inhibitor, preferentially inhibiting JAK1 and JAK3 [52].

- Tofacitinib in UC;

Tofacitinib (Xeljanz^®^, Pfizer, New York, NY, USA) has recently been approved in the United States and in Europe for the treatment of moderately to severely active UC [53,54]. The phase III randomized pivotal trial (OCTAVE Induction 1 and 2) evaluated tofacitinib’s efficacy at a dose of 10 mg twice daily vs. a placebo as an 8-week induction in moderately to severely active UC despite conventional therapy or therapy with anti-TNF-α [23]. Patients from OCTAVE Induction who had a clinical response (a decrease from the induction trial’s baseline in the total Mayo score of at least three points and at least 30%, with a decrease in the rectal bleeding subscore of at least one point or an absolute rectal bleeding subscore of 0 or 1) were eligible to participate in the randomized OCTAVE Sustain trial, which evaluated maintenance therapy with tofacitinib at a dose of 5 mg twice daily or 10 mg twice daily vs. a placebo for 52 weeks. A total of 1157 patients were included in the OCTAVE program, totaling 2404 patient-years of exposure. In both the Octave Induction 1 and 2 studies, remission at 8 weeks, defined as a total Mayo score of ≤2 with no individual subscore >1, and a null rectal bleeding subscore, was statistically more frequent in the tofacitinib group than in the placebo group (18.5% vs. 8.2% and 16.6% vs. 3.6% in the tofacitinib and placebo group, respectively). Interestingly, a subgroup analysis from the three OCTAVE trials showed that a clinical response (≥3 points and a ≥30% decrease in the Mayo total score from the baseline, plus a ≥1 point decrease in the rectal bleeding subscore or an absolute rectal bleeding subscore of 0 or 1) was attained in more than half (52.2%) of the primary non-responders (representing 42% of the total number of patients) 4 weeks after the end of a prolonged 16-week induction phase. Of note, in subgroup analyses, tofacitinib was more efficient than the placebo at inducing remission in patients who had prior anti-TNF-α treatment. In patients without prior anti-TNF-α treatment, the effect of tofacitinib on inducing remission was significant in OCTAVE Induction 2 but not in OCTAVE Induction 1.

A post-hoc analysis of OCTAVE Induction 1 and 2 at day 3 after induction reported a rapid clinical improvement in the tofacitinib group, illustrated by a significant reduction in the stool frequency (28.8% vs. 17.9%, *p* < 0.01) and rectal bleeding subscores (32.0 vs. 20.1%, *p* < 0.01) and in the total number of daily bowel movements compared with the placebo group [55].

Tofacitinib also demonstrated long-term efficacy in the OCTAVE Sustain trial. Remission at 52 weeks was 40.6% and 34.3% with the 10 mg dose and the 5 mg dose, respectively, which was significantly higher than in the placebo group (11.1%, *p* < 0.001) [23].

OCTAVE Open, an open-label long-term phase III extension, showed that remission persisted, 12 months after reducing tofacitinib from 10 mg to 5 mg twice a day, in 74.6% of the 66 patients who were still in remission after 52 weeks of treatment with the tofacitinib 10 mg bid. On the other hand, nearly half (49.1%) of the 57 patients who experienced treatment failure with maintenance with 5 mg of tofacitinib twice a day were in remission 12 months after escalating to 10 mg twice a day [56].

The results of the phase IIIB/IV double-blind randomized parallel group follow-up study evaluating remission after 6 months of treatment with tofacitinib 5 mg or 10 mg twice a day are expected to be published soon [57]. Tofacitinib is currently being evaluated in children aged 2 to 17 years old with moderately to severely active UC in an open-label induction and maintenance phase III study [58]. A phase III study investigating whether a diet intervention could help induce a clinical and biochemical response to tofacitinib therapy in UC is currently recruiting [59]. Regarding patient-reported outcomes, a phase II study of tofacitinib investigated the Inflammatory Bowel Disease Questionnaire (IBDQ) score as secondary endpoint. The IBDQ score did not significantly change with tofacitinib vs. a placebo, except with the 15 mg bid dosage, which was dropped for phase III studies [60]. Future studies considering tofacitinib through patient-reported outcomes and quality of life would be relevant.

Tofacitinib’s half-life is approximately three hours [61]. A study on tofacitinib’s pharmacokinetics showed that no significant change in tofacitinib exposure (apparent oral clearance) was seen over the duration of treatment. Additionally, age, sex, body weight, and baseline disease severity had no clinically significant impact on the apparent oral clearance and volume of distribution, indicating no need to adjust the dose of tofacitinib [62].

If the efficacy results of OCTAVE are promising for both induction and long-term remission in UC, some safety issues deserve further assessment. In the overall OCTAVE program (*n* = 1157), five patients experienced a serious thromboembolic event, including one leading to death. One patient had deep vein thrombosis (DVT) and four had pulmonary embolism (PE), representing an incidence rate of 0.04 (95% CI, 0.00–0.23) patients with events per 100 patient-years for DVT and an incidence rate of 0.16 (95% CI, 0.04–0.41) for PE. All patients with DVT or PE received an average total daily dose of ≥15 mg of tofacitinib and all had venous thromboembolism risk factors [63]. Curtis et al. estimated the incidence of venous thromboembolic events in patients treated with anti-TNF-α in a medico-administrative database. Venous thromboembolic events seemed to be more frequent with anti-TNF-α than with tofacitinib (0.16 (95% CI, 0.04–0.41) vs. 0.54 (95% CI, 0.30–0.89) for PE and 0.04 (95% CI, 0.00–0.23) vs. 1.41 (95% CI, 1.00–1.93) for DVT) [64]. Note that these results should be taken with caution as the two cohorts were not comparable; data about UC severity and the general medical history were not available in the medico-administrative database used by Curtis et al. Besides, these results are inconsistent with data observed from the long-term use of tofacitinib in rheumatoid arthritis: in a post-marketing study on 4369 patients with moderate to severe rheumatoid arthritis with at least one cardiovascular risk factor, tofacitinib intake at 10 mg twice daily was associated with an increased risk of pulmonary embolism and all-cause mortality compared with anti-TNF-α [65,66]. Surprisingly, these events do not seem to be dose-dependent. Indeed, in another study investigating the safety of tofacitinib in rheumatoid arthritis, the incidence rates of DVT were 0.17 per 100 patient-years of exposure (95% CI, 0.09–0.27) with 5 mg of tofacitinib twice daily and 0.15 (95% CI, 0.09–0.22) with 10 mg twice daily and the incidence rates of PE were 0.12 (95% CI, 0.06–0.22) with 5 mg of tofacitinib twice daily and 0.13 (95% CI, 0.08–0.21) with 10 mg twice daily [67]. If these results suggest that the risk of thromboembolism related to tofacitinib may differ between patients with rheumatoid arthritis or UC, the venous thromboembolic risk should be cautiously evaluated before initiating tofacitinib treatment in patients with UC.

Based on preliminary results of a randomized phase IIIB/IV safety trial of tofacitinib in rheumatoid arthritis patients, the FDA issued in February 2021 an alert on a possible increased risk of heart-related problems and cancer in patients with rheumatoid arthritis taking tofacitinib [65,68]. Studies published so far have not shown an increased risk of cancer associated with tofacitinib use in IBDs [64,69]. For example, Curtis et al. estimated an incidence rate of malignancies (excluding nomelanoma skin cancer (NMSC)) in UC patients of 0.63 per 100 patient-years (95% CI, 0.43–0.90) and 0.69 (95% CI, 0.40–1.11) with anti-TNF-α and tofacitinib, respectively [64]. Incidence rates of NMSCs were lower with tofacitinib (0.78 per 100 patient-years (95% CI, 0.47–1.22)) than with anti-TNF-α (1.69 (95% CI, 1.35–2.10)).

In all OCTAVE trials, a higher frequency of overall infections was observed with tofacitinib: 23.2% and 18.2% in the tofacitinib group vs. 15.6% and 15.2% in the placebo group for induction in OCTAVE 1 and 2, respectively; and 35.9% in the 5 mg tofacitinib group and 39.8% in the 10 mg tofacitinib group vs. 24.2% in the placebo group for maintenance [23]. However, only 2.9% of the patients developed serious infections and only 1.9% opportunistic infections [69]. Real-life populational cohort data suggest a lower risk of serious infections with tofacitinib (1.75 per 100 patient-years (95% CI, 1.27–2.36)) than with anti-TNF-α (3.33 (95% CI, 2.73–4.02)) [64]. However, a 10-fold increased risk of herpes zoster infections was identified with tofacitinib, especially with 10 mg of tofacitinib twice a day, particularly during the maintenance phase (5.1% vs. 0.5% with a placebo). Herpes zoster infections were all cutaneous except in three cases (one meningitis case and two ophthalmic cases) [23,69]. Curtis et al. estimated a higher incidence rate of herpes zoster with tofacitinib (3.57 events per 100 patient-years (95% CI, 2.84–4.43)) than with anti-TNF-α (1.77 (95% CI, 1.34–2.29)) [64].

A 61-week study showed that tofacitinib use may be associated with an increase in total cholesterol, high-density lipoprotein cholesterol, and low-density lipoprotein cholesterol, without significant changes in the LDL/HDL and total/HDL cholesterol ratios [56]. A creatine kinase level increase, not clinically meaningful, was observed in patients treated with tofacitinib [70].

The safety of long-term tofacitinib use must be carefully monitored and individual risk factors for adverse events (AEs) should be cautiously assessed before prescription.

- Tofacitinib in CD.

Tofacitinib has also been developed for CD. In the phase II trial vs. a placebo, tofacitinib failed to show a significant difference in clinical response (defined as a decrease in the Crohn’s disease activity index (CDAI) score of at least 100 points from the baseline) or in remission (CDAI score < 150) in the 8-week induction phase and in the maintenance of the response at 26 weeks in responders from the induction phase [24]. Moreover, in patients in remission at 26 weeks, a high discontinuation rate was observed (41.3%) during the open-label 48-week extension due to an insufficient clinical response and a high rate (50%) of infections [71]. Development of tofacitinib for CD was therefore interrupted.

Tofacitinib is a pankinase inhibitor with low selectivity, which may explain the adverse events encountered and its lack of efficacy in CD. Some more selective JAK inhibitors have been developed to improve their benefit/risk ratio.

#### 3.1.2. Upadacitinib

Upadacitinib (RINVOQ™, Abbvie, IL, USA) is a JAK1-selective inhibitor, recently approved for the treatment of rheumatoid arthritis, with a four-hour half-life [72].

- Upadacitinib in UC;

In an 8-week phase IIb induction study conducted in moderately to severely active UC, upadacitinib induced a dose-dependent remission (8.5% (*p* = 0.052), 14.3% (*p* = 0.013), 13.5% (*p* = 0.011), and 19.6% (*p* = 0.002) of patients for 7.5 mg, 15 mg, 30 mg, and 45 mg of upadacitinib once daily, respectively, compared with 0% in the placebo group) [25]. Interestingly, 73% of the patients had already been treated with anti-TNF-α and the analysis was adjusted for prior biologic use, baseline corticosteroid use, and baseline adapted Mayo score. The same effect was noticed for endoscopic improvement at 8 weeks (14.9%, 30.6%, 26.9%, and 35.7% for 7.5 mg, 15 mg, 30 mg, and 45 mg of upadacitinib, respectively, vs. 2.2% with a placebo). A clinical response at week 2, according to the partial Mayo score, was observed in up to 55.4% of the patients, suggesting a rapid onset of action of upadacitinib.

Upadacitinib did not seem to induce a higher rate of infections. One case of disseminated cutaneous-only herpes zoster (1.8%) was observed in a patient treated with 45 mg of upadacitinib. One patient dosed at 45 mg developed PE and DVT 26 days after treatment discontinuation, but he had known risk factors of venous thromboembolism. Some cases of anemia (<10%), hepatic disorders (transaminase elevations, 4–11% according to the dosage), increases in cholesterol levels, and increases in creatine phosphokinase levels (<10%) were observed in patients treated with upadacitinib. The phase III trial is being finalized, with two induction and maintenance phase III trials having completed their recruitment [73,74], and results are expected soon. A long-term phase III extension study following patients for up to 5 years is currently enrolling [75].

- Upadacitinib in CD.

A double-blind phase II trial evaluated the efficacy and safety of upadacitinib in patients with moderate to severe CD [26]. A total of 96% of the included patients had already been treated with at least one anti-TNF-α, resulting in an inadequate response or intolerance. Clinical remission at week 16 was observed in 13% of patients receiving a 3 mg dosage, 27% of patients receiving 6 mg (*p* < 0.1 vs. a placebo), 11% of patients receiving 12 mg, 22% of patients receiving 24 mg twice daily, and 14% of patients receiving 24 mg of upadacitinib once daily vs. 11% of patients receiving a placebo. The effect on clinical remission in CD was not compelling, except for the 6 mg dosage, but the number of patients treated with this dose was small (n = 37). The dose–response relationship was not significant. A statistically significant, but not clinically relevant, effect was observed on endoscopic remission with 24 mg of upadacitinib once or twice daily (14% and 22% of the patients, respectively). Four percent of the treated patients developed a serious infection. An open-label phase II extension study evaluating the efficacy and safety of upadacitinib at five years in patients with CD is currently ongoing [76]. Note that some extended-release formulations are in development to improve convenience for patients.

Two phase III studies evaluating the efficacy and safety of upadacitinib for the induction of remission are currently recruiting and are expected to be completed by the end of 2021 [27,77]. Concomitantly, an associated maintenance and long-term extension study is also open for enrolment [78].

In conclusion, intake of upadacitinib once daily seems to be of potential interest to induce remission in UC, but its long-term efficacy and safety deserve to be further investigated, particularly to see whether its selectivity profile will provide different results to tofacitinib. Evaluation of upadacitinib’s efficacy in CD is ongoing in a phase III trial, but the results from previous phases are not sufficiently consistent to conclude that there is a clear benefit.

#### 3.1.3. Filgotinib

Filgotinib (JYSELECA™, Gilead, Foster City, CA, USA) is also a JAK1-selective inhibitor, approved for use in rheumatoid polyarthritis. It has a six-hour half-life and is administered once daily [79].

- Filgotinib in UC;

Filgotinib has been investigated in moderate to severe UC. A randomized placebo-controlled phase IIB/III study, SELECTION1, has just been completed and enrolled 1348 patients [28]. The manufacturer’s communication reports that 200 mg of filgotinib was efficient in achieving clinical remission at Week 10 vs. a placebo in biologic-naive patients (26.1% vs. 15.3%, *p* = 0.02) [80]. In biologic-experienced patients, the effect of filgotinib on remission at Week 10 vs. a placebo was lower but still significant: 11.5% vs. 4.2%, *p* = 0.01. However, no comparison of filgotinib vs. biologics was made. A higher proportion of patients maintained remission through Week 58 vs. a placebo (37.2% of patients receiving 200 mg of filgotinib compared with 11.2% of patients treated with a placebo, *p <* 0.0001). The publication of full data is expected. A long-term extension study is currently ongoing [81].

- Filgotinib in CD.

FITZROY, a randomized, double-blind, placebo-controlled phase II trial, enrolled 174 patients with moderate to severe CD [29]. Patients recruited were either anti-TNF naive or anti-TNF failures. Filgotinib (200 mg) once a day induced remission after 10 weeks in 47% of the patients vs. 23% with a placebo (*p* = 0.0077). Filgotinib seemed to be more efficient at inducing remission in anti-TNF naive patients (60% vs. 13% with a placebo) than in anti-TNF-experienced patients (37% vs. 29% with a placebo). Only 3% of the patients reported serious infections. Too few patients (n = 44) were enrolled in the maintenance therapy study to draw any conclusion. A phase III study investigating filgotinib in the induction and maintenance of remission in CD is currently recruiting (DIVERSITY1) [82] and is associated with a long-term extension study (DIVERSITYLTE) [83]. A phase II study dedicated to patients with elective small bowel localization has been conducted in 78 patients, but the results are not available yet [84]. Of note is that, in toxicity studies with animal models, filgotinib induced germ cell depletion and testicular atrophy [85]. The FDA has requested data about the potential impact of filgotinib on sperm parameters before reviewing its New Drug Application in rheumatoid arthritis [86]. A phase II study investigating the testicular safety of filgotinib (in terms of a change in sperm concentration) in adult males with moderate to severe IBD is ongoing [87].

#### 3.1.4. Other JAK Inhibitors

SHR0302 is a highly selective JAK1 inhibitor that has been evaluated in UC patients [30]. The sponsor has announced positive results, but the data were not available in May 2021. A phase II study is currently recruiting to investigate SHR0302 in CD [31].

Peficitinib (Smyraf™, Astellas Pharma, Tokyo, Japan) is a pan-JAK inhibitor with an approximately 6- to 7-fold greater selectivity for JAK3 [88]. It has currently been approved in Japan for the treatment of rheumatoid arthritis. Peficitinib has been evaluated in moderate to severe UC in a phase IIb study [32], but no statistically significant effect on change in the Mayo score was observed at Week 8. To our knowledge, no other trial has been declared.

PF-06651600, a JAK3 inhibitor, and PF-06700841, a TYK2/JAK1 inhibitor (Pfizer, New York, NY, USA), have been investigated in moderate to severe UC in a phase IIb study [33], the results of which are not available yet. The two products will also be studied in CD in a phase IIa study that is currently recruiting [34].

Deucravacitinib (BMS-986165) is a selective TYK2 JAK inhibitor under development for psoriasis, with promising results [89,90]. Two phase II studies are currently recruiting in moderate to severe UC [35,91] and one phase II study is currently recruiting in moderate to severe CD [36]. These studies are not expected to be completed before 2023.

TD-1473 (Theravance Biopharma) is a gut-selective pan-JAK inhibitor. A phase IIB/III study is currently recruiting in moderately to severely active UC (RHEA [37,92]) and one phase II study in moderately to severely active CD (DIONE [38]).

Finally, OST-122 (Oncostellae S.L, Santiago de Compostela, Spain), a gut-restricted and subtype-selective JAK3/TYK2/Ark5 inhibitor, is currently being evaluated in a phase IB/II study in moderate to severe UC [39], with completion estimated to occur in the second quarter of 2022.

In conclusion, JAK inhibitors represent a promising therapeutic class for both the induction and maintenance of remission in UC and CD, with a rapid onset of action. However, differences in both safety and efficacy have been observed, probably based, at least partially, on differences in JAK selectivity. The serious adverse events raised in clinical trials (infections, malignancies, and cardiovascular and thromboembolic events) advocate for a long-term assessment of their safety. The individual risks and benefits should be cautiously weighted, and discussed with the patient, before initiating treatment. In the coming years, gut-restricted and selective JAK inhibitors may substantially improve the safety of this therapeutic class.

### 3.2. Sphingosine 1-Phosphate Receptor Modulators

Sphingosine 1-phosphate (S1P) is a membrane-derived lysophospholipid signaling molecule acting as an extracellular lipid mediator via extracellular activation of five different subtypes of G-protein-coupled receptors (S1PR1–5) [93,94]. S1P receptors (S1PRs) have different distributions. While subtypes 2 and 3 may be linked to cardiovascular, pulmonary, and cancer-related adverse events, subtypes 1, 4, and 5 are involved in regulation of the immune system. Specifically, S1PR1 prevents lymphocytes from exiting the lymph node and being trafficked to inflamed tissues, thus modulating immunity [95]. These effects are reversed upon withdrawal of the agent [94].

- Ozanimod is an orally administered small molecule that selectively targets the sphingosine 1-phosphate receptor subtypes 1 and 5 [96]. Ozanimod was investigated in a phase II clinical trial, TOUCHSTONE, in 197 adults with moderate to severe UC who were randomized to receive either a placebo, ozanimod 0.5 mg, or ozanimod 1 mg once daily, with a dose escalation during the first week (Table 1) [40]. At week 8, ozanimod 1 mg once daily was associated with a slightly higher rate of clinical remission (Mayo Clinic Score (MCS) ≤ 2 with no subscore > 1) than the placebo (16% vs. 6%, *p* = 0.048). The open-label extension of this study included 170 patients (86%) who were switched to or continued to receive ozanimod 1 mg once daily [97]. During the first year, 28% of the patients discontinued treatment and the discontinuation rates were 15% to 18% annually through years 2–4. The clinical remission rate, including the non-responder imputation, was 54.7% at week 56 and 36.5% at week 200, while the clinical response rate (a decrease in the MCS of ≥3 points and ≥30% and a decrease in the rectal bleeding subscore of ≥1 point or a subscore ≤ 1) was 71.2% and 41.2% at week 56 and 200, respectively [97]. Histologic remission, an endoscopic improvement, endoscopic remission, and mucosal healing at week 56 were achieved in 18.2%, 22.9%, 5.9%, and 2.4% of the patients, respectively. The most common adverse events were hypertension (5.9%), upper respiratory infection (5.9%), lymphopenia (5.3%), and elevated gamma glutamyltransferase (5.3%).

In CD, ozanimod was investigated in a phase II uncontrolled prospective observer-blinded endpoint (PROBE) trial, STEPSTONE, including 69 patients with moderate to severe CD (Table 1) [41]. Patients received an escalating dose of treatment during a week to reach a dose level of ozanimod 1 mg once daily. At week 12, 23.2% (95% CI 13.9–34.9) had an endoscopic response, 10.1% (95% CI 4.2–19.8) had endoscopic remission, 56.5% (95% CI 44.0–68.4) had a clinical response, and 39.1% (95% CI 27.6–51.6) had clinical remission. The most commonly serious adverse events reported were complications of CD (9%) and abdominal abscesses (3%). It is not possible to draw a conclusion on the therapeutic interest in the absence of a control group. A phase III clinical trial and an open-label long-term efficacy and safety trial are currently ongoing [98,99,100,101].

- Etrasimod is an orally administered S1P modulator selectively targeting S1P receptor subtypes 1, 4, and 5 [95]. Etrasimod was investigated in the phase II OASIS randomized, parallel-group induction trial, which included 156 patients with moderate to severe UC [42] (Table 1). At week 12, the clinical improvement (a change from the baseline in the three-component Mayo Clinic Score) was higher in patients treated with 2 mg of etrasimod compared with the placebo (difference, 0.99 points; 90% CI 0.30–1.68) as well as the endoscopic improvement (41.8% vs. 17.8% for the placebo; *p* = 0.003). In the open-label extension study including 118 patients receiving 2 mg of etrasimod daily up to week 52, 64% of patients had a clinical response, 33% had clinical remission, and 43% had an endoscopic improvement [102]. Patients who achieved a clinical response, clinical remission, or an endoscopic improvement at week 12 maintained the effect of the treatment with 85%, 60%, and 69% of patients with a clinical response, clinical remission, or an endoscopic improvement, respectively, at the end of treatment. Steroid-free clinical remission was observed in 22% of the patients. A total of 60% of the patients experienced treatment emergent adverse events (TEAEs), the most common being worsening of UC (19%) and anemia (11%). When excluding patients with worsening of UC, 51% of the patients (46/91) experienced a TEAE and three patients experienced a serious adverse event [102].

### 3.3. Other SMDs

#### 3.3.1. Anti-Integrins

Anti-integrins aim at interfering with immune cell trafficking, especially leucocytes. Integrins are heterodimeric glycoproteins with one α sub-unit and one β sub-unit, with several structural isoforms, that recognize and bind cell adhesion molecules (CAMs). These integrins include α4β1 (present in most leukocytes) and α4β7, which is found specifically on lymphocytes in the digestive tract. Anti-α4β1 integrins were first considered as a therapeutic option with natalizumab [103]. However, the occurrence of progressive multifocal leukoencephalopathy precluded its use in IBD patients, which can be explained by α4β1′s function of mediation of lymphocyte trafficking in the gut and brain [104]. α4β7 inhibition, on the contrary, was found to be more successful with vedolizumab and etrolizumab in both CD and UC.

PN-943 (Protagonist Therapeutics, Inc., Newark, CA, USA) is an orally administered, gut-restricted peptide targeting MAdCAM1 [105]. PN-943 was well tolerated in a phase I trial in eight patients, with a 94% blood receptor occupancy, after two weeks of daily administration at a 1000 mg dose (data from the manufacturer). The phase II trial is ongoing, and inclusions are expected to be finished in June 2022. Another more advanced candidate, PF-00547659 (ontamalimab), also prevents the link between MAdCAM1 and the α4β7 integrin [106]. The phase II trial (TURANDOT), a 12-week treatment, included 357 patients (152 anti-TNF-α naive and 205 anti-TNF-α experienced) with active UC (total Mayo score ≥ 6 with endoscopic score ≥ 2) (Table 1) [43]. Patients were randomized to be administered 7.5, 22.5, 75, or 225 mg of PF00547659 or a placebo. Remission (total Mayo score ≤ 2 with no subscore > 1) was significantly higher at 12 weeks in the three lowest active groups (11.3%, 16.7%, and 15.5%, respectively) compared with 2.7% in the placebo group. Interestingly, remission in the same active groups was markedly higher in anti-TNF-α naive than in anti-TNF-α experienced patients (16.7–23.3% vs. 7.3–9.8%). Patients who completed the 12 weeks of treatment were subsequently included in the ongoing, 144-week open-label extension trial TURANDOT II to assess the long-term safety of PF-00547659. Conversely, PF-00547659 failed to demonstrate efficacy in CD [107]. The OPERA trial, a randomized, double-blind, placebo-controlled phase II study, included moderate to severe CD patients (CDAI range: 220–450) who failed immunosuppressive drugs or anti-TNF-α [44]. No difference in remission (CDAI reduction > 70 points at week 8 and week 12 compared with the baseline) was observed between the placebo and treatment groups on the main outcome (week 8: 16.7% in remission in the placebo group compared with 29.1%, 23.8%, and 26.9% of those in the PF-00547659 22.5 mg, 75 mg, and 225 mg groups, respectively; at week 12: 23.0%, 26.8%, 28.5%, and 29.6% of patients in these respective groups were in remission).

#### 3.3.2. Phosphodiesterase 4 Inhibitors

Phosphodiesterase 4 (PDE4) is an intracellular enzyme that degrades the cyclic AMP (cAMP) in many cells, including immune cells, macrophages, and T cells. This catabolism promotes numerous pro-inflammatory cytokines by activating the nuclear transcription factor kappaB (NF-κB) and, at the same time, lowers the production of anti-inflammatory messengers [108]. However, no drug candidate has passed the phase II stage yet (Table 1) [45]. Apremilast (Otezla, Celgene^®^, Summit, NJ, USA), a PDE4 inhibitor approved for use in psoriatic arthritis and Behçet’s disease, has been evaluated in a phase II, double-blind, placebo-controlled RCT in UC [46]. After 12 weeks, patients treated with 30 mg of apremilast showed a higher clinical remission rate, defined as a total Mayo score of 2 with no individual subscore > 1, than patients treated with a placebo (31.6% vs. 12.1% in the placebo group, *p* = 0.01). Surprisingly, 40 mg of apremilast did not reach statistical significance (21.8% vs. 12.1% for the placebo, *p* = 0.27). Mucosal healing at 12 weeks, defined as a Mayo endoscopic score (MES) of 1 and a Geboes score of < 2, showed similar results, with only the 30 mg group showing efficacy (33% vs. 15.5% for the placebo, *p* = 0.03). Overall, treatment was well tolerated, with only one case of pancreatitis in a patient treated with 40 mg with a history of hepatobiliary disease. The most frequent AEs were headache and nausea (21.1% and 5.3%, respectively, in the 30 mg group and 25.5% and 10.9%, respectively, in the 40 mg group).

#### 3.3.3. SMAD7 Blockers

Targeting the SMAD as a therapeutic pathway in IBDs goes upstream from the other drugs. SMAD is an intracellular protein that, once phosphorylated, inhibits the anti-inflammatory activity of transforming growth factor-β1 (TGF-β1). SMAD7, in particular, plays a key role in this process, and SMAD7 blockers aim at restoring the anti-inflammatory activity of TGF-β1 [109,110]. Mongersen (GED-0301; Celgene^®^) is an antisense oligonucleotide that prevents the transduction of SMAD7 protein and consequently increases TGF-β1 activity. Mongersen was first evaluated in a phase II, double-blind, placebo-controlled RCT testing three doses (10, 40, and 160 mg) in moderate to severe CD patients (Table 1) [47]. Of the 160 analyzed patients, clinical remission (defined as a CDAI score of <150 at day 15 and maintenance of a CDAI score of <150 until day 28) was significantly more frequent for the two highest doses (55% and 65% for the 40 mg and 160 mg doses, respectively) than with the placebo or the 10 mg dose (10% and 12%, respectively). A response was reached from day 15 for 65% of the patients receiving 160 mg per day. Nine SAEs (5.6%) occurred and were mostly related to disease worsening. However, the subsequent phase III trial was prematurely halted after 78.6% (551/701) of the patients completed the study as the clinical remission rate at 12 weeks was similar between Mongersen and the placebo (22.8% vs. 25.0%, *p* = 0.621). Development of Mongersen was definitively stopped [111].

#### 3.3.4. Other Small Drugs

IMU-838 (vidofludimus calcium, Immunic Therapeutics Inc., New York, NY, USA) is a new compound that selectively inhibits the human enzyme dihydroorotate dehydrogenase (DHODH) [112]. DHODH plays a key role in pyrimidine synthesis and is expressed at high levels in proliferating or activated lymphocytes. Thus, IMU-838 is able to target activated and rapidly proliferating lymphocytes by inhibiting the DHODH. This leads to a reduction in pro-inflammatory cytokine release, including interleukin IL-17 (A and F) and interferon gamma (IFNγ), and to an increased apoptosis of activated lymphocytes. Vidofludimus calcium has shown a safety profile comparable to that of a placebo in rheumatoid arthritis and multiple sclerosis studies, and the double-blind, placebo-controlled phase II trial (CALDOSE-1) is ongoing and is expected to finish in 2022 (Table 1) [48,113,114,115].

ABX464 (Abivax, Paris, France) is a novel, promising, and orally administered SMD that was developed for UC and HIV disease due to its concomitant antiviral and anti-inflammatory properties [49]. ABX464 targets the Cap Binding Complex (CBC), a complex intervening in the transcription process [116]. Once bound to the CBC, ABX464 upregulates the expression of miR-124, a protein with anti-inflammatory properties. In a phase IIa randomized, placebo-controlled, and double-blind 8-week induction study including patients < 75 years old with moderate to severe UC, 35.0% and 70.0% of patients in the active group achieved clinical remission and clinical response vs. 11.1% and 33.3% in the placebo group, respectively (Table 1) [50]. At 2 years, 69% (11/16) of the patients experienced clinical remission with 50 mg ABX464, and 44% (7.16) had total endoscopic remission at the same time. The subsequent open-label trial expecting 30 patients is estimated to be terminated in summer 2021 [117].

Finally, BT-11 is an orally administered, gut-restricted, and first-in-class small molecule that targets the lanthionine synthetase C-like 2 (LANCL2) pathway and immunometabolic mechanisms. Preclinical models revealed that administration of BT-11 leads to a decrease in neutrophils, T-helper type-1 cells, TNF-α, IFNγ, and MCP1 expression in the colon. The phase I trial validated the safety profile of BT-11, and phase II studies are ongoing in CD and UC patients (Table 1) [51,118].

## 4. Conclusions: What Should Be the Future for SMDs

SMDs will come to occupy an even larger share of the IBD pharmacological treatments as evidence of their efficacy keeps being published. The lack of immunogenicity, the comfort of an orally administered compound, and the rapid onset of action justify their increasing use in the treatment of UC and CD patients and could enhance the medication adherence. Nevertheless, the place of SMDs in the therapeutic path deserves to be better defined. If placebo-controlled trials remain the gold standard from health agencies’ point of view, it only adds another authorized drug to the already abundant queue of therapeutic options and will no longer be a hot topic. Comparing the new SMDs with the current arsenal should now be the priority so that physicians can give the best treatment to their patients. Head-to-head phase III trials, such as the HIBISCUS I and II trials in UC and the GARDENIA trial in CD comparing etrolizumab to anti-TNF-α, are ongoing. The results of these trials may change the therapeutic paradigm and the treatment recommendations. Additionally, if a step-up strategy remains the standard of care, a top-down strategy could be considered in patients with severe disease and poor prognostic factors, who could be treated with SMDs as a first-line treatment.

Currently, SMDs cannot be considered a panacea, as illustrated by the mean remission rates of 40% observed in clinical trials, but their full potential will only be perceived in a few years, with the accumulation of real-world data. The combination of anti-TNF-α with conventional treatments was first evaluated by post-marketing experience. Real-world feedback on the use of a combination of biological drugs and SMDs is already emerging [119]. Nevertheless, the evaluation will probably be more complex and challenging than with anti-TNF-α due to the multiplicity of mechanisms of action of SMDs. Their efficacy in patients previously treated with anti-TNF-α and in patients with mild IBD also deserves to be further investigated.

From the patient point of view, the oral formulation is certainly a major improvement in the quality of life and adhesion to treatment. It could also represent substantial savings in terms of healthcare costs for society, avoiding the required hospitalizations for intravenous treatments. Medico-economic evaluations of SMDs are currently limited to studies on rheumatologic autoimmune disorders [120,121], and dedicated cost-effectiveness studies in patients with IBD are required, since SMDs are relatively costly. For example, the wholesale acquisition cost ranges from $75 per unit for tofacitinib to $163 for upadacitinib [122].

Finally, the recent FDA publication on the potential cardiac and vascular effect of tofacitinib reminds us that the safety of recently developed molecules needs close and careful monitoring. Furthermore, it would be worth paying greater attention to the efficacy and safety of SMDs through patient-reported outcomes in future trials. Some studies suggested that patient-reported IBD symptoms have a huge impact on quality of life, even in patients who are in remission according to their physicians [123]. In conclusion, SMDs are a promising alternative to anti-TNF-α treatments and present many advantages that will possibly make them outdo anti-TNF-α treatments in the near future, assuming that no serious safety issues occur as their duration of follow-up increases.

## Figures and Tables

**Table 1:** Main published and ongoing clinical trials with Small Molecules Drugs (SMDs) in inflammatory bowel disease.

Type of SMD	Phase, Indication	Design and Intervention	Main Results
JAK inhibitors			
Tofacitinib (pankinase, JAK1, JAK3)	III, UC (OCTAVE PROGRAM) [23]	OCTAVE-1 and OCTAVE-2 trials: an 8-week induction trial in 598 and 541 patients with moderate to severe disease, respectively, naive or biologic-experienced 10 mg/placebo (4:1)	Higher clinical remission in the active group than in the placebo group (OCTAVE-1: 18.5% vs. 8.2%, *p* = 0.007; OCTAVE-2: 16.6% vs. 3.6%, *p* < 0.001)
		A 52-week sustained trial for 593 responders from OCTAVE induction trials; 3 arms: 10 mg/5 mg/placebo (1:1:1)	Higher clinical remission in the 10 mg group vs. the placebo group (40.6% vs. 11.1%, *p* < 0.001), but not in the 5 mg group (34.3% vs. 11.1%)
	IIb, CD [24]	An 8-week induction trial in 180 patients with moderate to severe disease, naive or biologic-experienced 10 mg/5 mg/placebo (1:1:1)	No significant improvement in remission (43.0%/43.5%/36.7%, all tests vs. placebo NS)
		A 48-week OLE trial in 150 patients 10 mg/5 mg (1:1)	Last observation carried forward 85.5% in 10 mg/39.8% in 5 mg
Upadacitinib (JAK1)	IIb, UC (U-ACHIEVE) [25]	An 8-week induction trial in 250 patients with moderate to severe disease, naive or biologic-experienced 45/30/15/7.5 mg/placebo (1:1:1:1:1)	A higher remission rate in all active groups vs. the placebo group except for the 7.5 mg group (19.6%/13.5%/14.3%/8.5%/0%)
	II, CD (CELEST) [26]	A 16-week induction trial in 220 patients with moderate to severe disease, naive or biologic-experienced 24 mg qd/24 mg bid/12 mg bid/6 mg bid/3 mg bid/placebo (1:1:1:1:1:1)	Significant clinical remission only for upadacitinib 6 mg bid vs. placebo 14%/22%/11%/27% (*p* < 0.1)/13%/11%
	III, CD (CELEST OLE) [27]	An OLE trial for patients who completed the (52-week) CELEST induction trial	Ongoing, Estimated Primary Completion Date: 2025
Filgotinib (JAK1)	III, UC [28]	SELECTION-1: a 10-week induction trial in 659 patients with moderate to severe biologic-naive disease and in 689 biologic-experienced patients 200 mg/100 mg/placebo (2:2:1)	Efficacy (clinical remission) of 200 mg filgotinib vs. placebo in both biologic-naive (26.1%vs. 15.3%) biologic-experienced patients (11.5% vs. 4.2%)
	II, CD [29]	FITZROY: 10-week induction in 172 patients with moderate to severe CD, naive or biologic-experienced 200 mg vs. placebo (3:1)	A higher remission rate in 200 mg filgotinib (47% vs. 23%, *p* < 0.001)
SHR0302 (JAK1)	II, UC [30]	An 8 to 12-week dose finding and efficacy trial in 164 patients with (expected) moderate to severe disease (3 doses vs. placebo)	Study completed in February 2021; results not published yet
	II, CD [31]	A 12-week dose finding and efficacy trial in 144 patients with (expected) moderate to severe disease (3 doses vs. placebo)	Ongoing, estimated study completion date: October 2021
Peficitinib (pankinase inhibitor, JAK3+)	IIb, UC [32]	An 8-week induction trial in 219 patients with moderate to severe disease 150 mg qd/75 mg bid/75 mg qd/25 mg qd/placebo (1:1:1:1:1)	No significantly higher response compared with placebo (27.3%/15.9%/15.9%/15.9%/7.0%)
PF-06651600 (JAK3) and PF-06700841 (TYK2/JAK1)	IIb, UC [33]	An 8-week dose ranging trial in 319 patients with (expected) moderate to severe disease, naive or biologic-experienced (3 doses PF-06651600/3 doses PF-06700841/placebo)	Study completed in May 2021; results not published yet
	IIa, CD [34]	A 12-week efficacy trial in 250 patients with (expected) moderate to severe disease, naive or biologic-experienced PF-06651600 (200 mg for 8 weeks + 50 mg for 4 weeks)/PF-06700841 (60 mg for 12 weeks)/placebo	Ongoing, estimated study completion date: June 2021
Deucravacitinib (TYK2)	II, UC [35]	A 12-week efficacy trial in 50 patients with (expected) moderate to severe disease, naive or biologic-experienced (2 doses vs. placebo)	Ongoing, estimated study completion date: April 2023
	II, CD [36]	A 12-week efficacy trial in 240 patients with (expected) moderate to severe disease, naive or biologic-experienced (2 doses vs. placebo)	Ongoing, estimated study completion date: March 2024
TD-1473 (gut-selective pankinase inhibitor)	II-III, UC [37]	An 8-week dose ranging phase II trial (3 dose groups or placebo) in 240 patients with moderate to severe disease, naive or biologic-experienced, followed by a phase III 8-week induction trial in 660 patients	Ongoing, estimated study completion date of phase II: July 2021
	II, CD [38]	A 12-week efficacy and safety trial in 160 patients with moderate to severe disease; 3 arms (2 doses and placebo)	Ongoing, estimated study completion date: August 2022
OST-122 (gut-selective JAK3/TYK2/ARK5)	I-II, UC [39]	A 28-day safety trial in 32 patients with moderate to severe disease; 3 arms (low dose, high dose, placebo)	Ongoing, estimated study completion date: April 2022
S-1-P receptor modulators			
Ozanimod S1PR1, S1PR5)	II, UC (TOUCHSTONE) [40]	An 8-week induction trial in 197 patients with moderate to severe disease, naive or biologic-experienced 1 mg/0.5 mg/placebo (1:1:1)	A higher clinical remission rate in the 1 mg group vs. the placebo group (16% vs. 6%, *p* = 0.048)
	II, CD (STEPSTONE) [41]	An uncontrolled trial in 69 patients with moderate to severe disease, naive or biologic-experienced 1 mg ozanimod for 11 weeks after a 7-day dose escalation	39.1% (95% CI 27.6–51.6) achieved clinical remission (CDAI < 150) and 56.5% (95% CI 44.0–68.4) exhibited a clinical response (CDAI decrease from baseline ≥100)
Etrasimod (S1PR1)	II, UC (OASIS) [42]	A 12-week induction trial in 156 patients with moderate to severe disease, naive or biologic-experienced; 3 arms (2 mg/1 mg/placebo) (1:1:1)	A significantly higher response rate (improvement in modified MCS from baseline) in the 2 mg group vs. the placebo group (+0.99, 95% CI 0.30–1.68)
Anti-integrins			
Ontamalimab (MAdCAM1)	II, UC (TURANDOT) [43]	A 12-week induction trial in 357 patients with moderate to severe disease, naive or biologic-experienced; 5 arms (225 mg/75 mg/22.5 mg/7.5 mg/placebo) (1:1:1:1:1)	A higher remission rate in all active groups than in the placebo group except for the 225 mg group (5.7%/15.5%/16.7%/11.3%/2.7%)
	II, CD (OPERA) [44]	A 12-week induction trial in 223 patients with moderate to severe disease, naive or biologic-experienced; 4 arms (225 mg/75 mg/22.5 mg/placebo) (1:1:1:1)	No significant difference in the remission rate at 12 weeks compared with placebo (29.6%/28.5%/26.8%/23.0%
PPD4 inhibitors			
Tetomilast (PDE4)	II, UC [45]	An 8-week induction trial in 186 patients with mildly to moderately active disease, biologic-naive. 3 arms: 50 mg/25 mg/placebo (1:1:1)	No statistical difference in success rates (improvement as defined by a reduction in DAI>3 at week 8) between the active groups and the placebo group (21%/16%/7%)
Apremilast (PDE4)	II, UC [46]	A 12-week induction trial in 170 patients with moderate to severe disease, biologic-naive. 3 arms: 40 mg/30 mg/placebo (1:1:1)	Clinical remission was significantly higher than the placebo group for the 30 mg group (31.6% vs. 12.1%), but not for the 40 mg group (21.8%)
SMAD7 blockers			
Mongersen	II, CD [47]	A 2-week induction trial in 166 patients with moderate to severe disease, biologic-naive. 4 arms: 160 mg/40 mg/10 mg/placebo (1:1:1:1)	Clinical remission was significantly higher in the active groups compared with the placebo group, except for the 10 mg group (65%/55%/12%/10%)
Other SMDs			
Vidofludimus calcium (DHODH)	II, UC (CALDOSE-1) [48]	A 10-week dose finding trial in 240 patients with (expected) moderate to severe disease, naive or biologic-experienced; 4 arms: 10 mg/30 mg/45 mg/placebo	Ongoing, Estimated Primary Completion Date: July 2022
ABX464 (microRNA-124)	II, CD [49]	A 16-week safety and efficacy trial in 30 patients with (expected) moderate to severe disease, naive or biologic-experienced; 2 arms: 50 mg/placebo	Ongoing, Estimated Primary Completion Date: July 2021
	IIa, UC [50]	An 8-week induction trial in 29 patients with moderate to severe disease, naive or biologic-experienced; 2 arms: 50 mg/placebo	No significant difference between the active group and the placebo group in clinical remission at 8 weeks (35.0% vs. 11.1%)
BT-11 (LANCL2)	II, UC [51]	A 12-week efficacy and safety trial in 198 patients with (expected) mildly to moderately active disease, naive or biologic-experienced; 3 arms: 1000 mg/500 mg/placebo	Study completed in May 2021; results not published yet

CD, Crohn’s Disease; CDAI, Crohn’s Disease Activity Index; CI, Confidence interval; JAK, Janus Kinase; MCS, Mayo Clinic Score; OLE, open label extension; UC, Ulcerative Colitis.

## Data Availability

Not applicable.
